# Retrospective Cohort Study of Low‐Value Hysterectomy Before and After Publication of the National Heavy Bleeding Clinical Care Standard in Regional Victoria

**DOI:** 10.1111/ajr.70049

**Published:** 2025-04-25

**Authors:** Natasha Daureen Frawley, Madison Phung, Benjamin Harrap

**Affiliations:** ^1^ Grampians Health Ballarat Victoria Australia; ^2^ Rural Clinical School, The University of Melbourne Ballarat Victoria Australia; ^3^ Department of Obstetrics and Gynaecology Deakin University Ballarat Victoria Australia

**Keywords:** heavy menstrual bleeding, hysterectomy, low‐value surgery, regional

## Abstract

**Objective:**

To evaluate the prevalence of low‐value care hysterectomy before and after publication of the National Heavy Menstrual Bleeding Clinical Care Standard (HMB Standard) in a regional Victorian hospital. The secondary aim was to assess whether compliance with the HMB Standard improved.

**Methods:**

Retrospective cohort design. All patients booked for a planned benign hysterectomy were included. Manual chart review was undertaken for demographics, surgical planning, procedure, and outcomes to 28 days.

**Design Setting:**

A single regional Victorian hospital within an area identified to be high volume for benign hysterectomy.

**Participants:**

Patients who planned benign hysterectomy in the 10 months prior (Group 1—Control) and 10 months after (Group 2—Post‐intervention) publication of the HMB Standard in October 2017.

**Main Outcome Measures:**

Low‐value hysterectomy was defined as the proportion of benign hysterectomies performed via the abdominal route in the absence of cancer or a previous caesarean section.

**Results:**

There were 64 patients in Group 1 and 60 in Group 2 included. Low‐value hysterectomy proportion had a non‐significant change from 9.4% in Group 1 to 11.7% in Group 2, 95% confidence interval = [−0.1303, 0.0857]. Compliance to the HMB Standard had mixed results.

**Conclusions:**

There was no clinically significant change in low‐value hysterectomy in the 10 months following publication of the HMB Standard, compared to 10 months prior, in a regional Victorian hospital. Uptake of therapeutic alternatives to hysterectomy was low.

**Summary**

- What is already known on this subject
  ○ Benign hysterectomy is a common, morbid procedure, and the Australian Atlas Report has identified variation in rates as an area of concern.
  ○ Non‐urban populations and those with socio‐economic disadvantage are more likely to have benign hysterectomy rather than non‐hysterectomy alternatives as treatment.
  ○ The three locations identified as having the highest rates, up to 6.6 times those with the lowest rate, in Australia, are all located in the Grampians region of Victoria. The reasons behind the high rate of benign hysterectomy for people living in the Grampians are not immediately apparent.
  ○ It is important to reduce low‐value hysterectomy as it may confer little patient benefit, or even cause harm. There are also equity implications with more disadvantaged patients potentially identified to have less access to non‐invasive treatments.
- What this paper adds
  ○ Our retrospective cohort study identified an insignificant increase in low‐value hysterectomies in a Victorian regional hospital in the 10 months after publication of the Australian Heavy Menstrual Bleeding Clinical Standard, compared to the 10 months prior. Due to the small sample size and the fact that methods did not limit for confounding factors, we cannot make any causal inference.
  ○ There was low uptake of therapeutic alternatives to hysterectomy, as identified by auditing compliance to the Heavy Menstrual Bleeding Clinical Care Standards, including low rates of endometrial ablation and the levonorgestrel intrauterine device prior to planned hysterectomy for benign heavy bleeding. This is important as it suggests that less invasive options that were promoted by the Clinical Care Standard may not have been thoroughly explored before progressing to low‐value surgery across both groups.
  ○ Future improvement work could therefore focus on strategies to increase access and uptake of therapeutic alternatives to hysterectomy in patients with benign heavy bleeding in this regional setting.

## 1 Introduction

Benign hysterectomy, defined as surgical removal of the uterus for a non‐cancerous indication [1], has particularly high rates in Australia [2, 3]. It is morbid, carrying a 7% and 9% risk of major and minor complications, respectively [4]. The most common indications in Australia are severe prolapse, pain, and abnormal uterine bleeding [3]. For many patients with these conditions, medical or less invasive surgical options exist, which are therapeutic alternatives to hysterectomy [5, 6]. Low‐value care is defined as an intervention where evidence suggests it confers little or no benefit to the patient, or where harm exceeds likely benefit [7, 8]. In the context of hysterectomy, Choosing Wisely Canada has identified that hysterectomy should only be offered in the context of heavy bleeding only if levonorgestrel intrauterine device (IUD) has been offered and declined [9]. Low‐value hysterectomy has been defined in previous research as benign hysterectomy using the abdominal approach and not associated with caesarean section or cancer [10, 11].

The Australian Atlas of Healthcare Variation reports identified that benign hysterectomy variation was an area of concern in both 2015 and 2017 [12]. Notably, the rates of benign hysterectomy in Australia varied markedly depending on where people live and tended to increase with socio‐economic disadvantage and in non‐urban populations [12]. The benign hysterectomy rate in 2017 was 6.6 times higher in the area with the highest rate compared to the area with the lowest rate [12]. The three locations identified as having the highest rates in Australia are all located in the Grampians region of Victoria. The reasons behind the very high rate of benign hysterectomy for people living in the Grampians are not immediately evident; however, several factors have been proposed, such as patient preference, education, practitioner preferences, training, patient insurance status, and socio‐economic demographic factors [12, 13].

Since the Australian Atlas report in 2017, the Australian Commission on Safety and Quality in Health Care published the Heavy Menstrual Bleeding Clinical Care Standard (HMB Standard) [14], and updated this in 2024 [15], to help mitigate inconsistencies in care. The guideline outlines eight standards, and standards 1–6 relate to General Practice care and standards 7–8 pertain to specialist gynaecologist care [16]. Hysterectomy is not recommended in the HMB Standard, unless other, less invasive treatments are considered inappropriate [14, 15].

The primary aim of this study was therefore to evaluate the prevalence of low‐value hysterectomy 10 months before and 10 months after the publication of the HMB Standard in a single major high‐volume Victorian referral hospital within the Grampians region. The secondary aim was to assess whether compliance with the HMB Standard across the eight recommended standards improved in the 10 months following its publication, compared to the 10 months prior. We hypothesised that low‐value hysterectomy would decrease and compliance to the HMB Standard would increase over the study period.

## 2 Methods

This is a retrospective cohort study. Research Ethics approval was obtained by the local hospital ethics department.

The setting was a single regional Victorian hospital with 200 inpatient beds, servicing a population of 250 000 people and 110 km from the closest tertiary centre. There are 260–300 elective gynaecological surgeries performed per annum, and there is good support from anaesthetics, urology, general surgery, as well as on‐site ICU, blood bank, and massive transfusion access.

The study participants were all patients who entered the public surgical waitlist for a benign hysterectomy at a single large regional Australian hospital within the Grampians region, Victoria, from 1 January 2017 to 31 August 2018 (20 months). This time period was selected as it covers 10 months before (Group 1: 1 January 2017–31 October 2017) and 10 months after (Group 2: 1 November 2017–31 August 2018) publication of the HMB Standard. The publication of the HMB Standard and Atlas report results was consistently discussed within the departmental gynaecology morbidity and mortality meetings, attended by a large proportion of consultants and registrars booking hysterectomy cases.

Study participants were identified manually by the lead gynaecology clinical nurse as patients were booked onto the surgical waitlist from clinic for a hysterectomy during the above dates; this was captured in the nurse's routine clinic audit. This process was checked by the gynaecology surgical booking liaison nurse against the electronic hospital surgical database using the search terms “hysterectomy,” “TLH,” “VH,” “TAH,” to check whether hysterectomy was performed.

Exclusion criteria were private patients due to incomplete data, for example, there was no referring letter or admission note detailing prior treatments. Excluded also were patients booked for hysterectomy, but on the operating day, a hysterectomy was not performed, as it was deemed not required, for example, for prolapse.

Data were collected by two authors independently, both manually and by via reviewing patient hospital records, including referral letters, investigations, operation reports, anaesthetic records, admission notes and histopathology results. Both authors were not blinded to the study aims. A standard data collection form was used. Disagreements about data were reviewed together and consensus reached. Quality of the data collection was ensured by having the second author perform data collection and check disagreements. No inter‐rater reliability was calculated.

Data collected included: patient demographics; indication for hysterectomy, other treatments offered; consent documentation; hysterectomy mode; anaesthetic time; histopathology; patient wait time; and composite major adverse outcome events (defined as return to theatre, readmission or representation to the Emergency Department within 28 days, Intensive Care Unit admission, unexpected blood transfusion and mortality outcome). Indications for hysterectomy were recorded using the FIGO PALM COEIN classification [17].

Assessment of low‐value hysterectomy, defined as abdominal hysterectomy in the absence of a history of caesarean section, was reported as a percentage of all benign hysterectomies, excluding those with incidental cancer on pathology. This number was calculated by dividing the number of people having an abdominal route for benign hysterectomy (in the absence of prior caesarean section) by the total number having benign hysterectomy.

The data were screened to review those who had hysterectomy with a primary or secondary indication of HMB. For these, compliance to the eight clinical care standards in the HMB Standard was assessed [12]. Results were binary.

We applied descriptive analysis to determine if compliance with these standards changed after the implementation of the HMB Standard.

Missing data were present in a small group of patients who had their hysterectomy contracted out to another hospital and therefore were removed from subsequent analysis including the composite major adverse outcome event. Negative status was assumed for missing binary outcomes in otherwise complete notes, such as being offered endometrial ablation or being screened for anaemia.

Data were analysed using Microsoft Excel, R version 4.0.5, and RStudio version 2022.07.2 + 576. Confidence intervals (CIs) were calculated using the Wilson score interval without a correction for continuity, calculating CI for the difference of proportions with a Z value of 1.96 corresponding to a 95% CI [18]. *p* value was calculated for the primary outcome using the *Z*‐test distribution formula for the difference in proportion. Chi‐square test was used to assess the *p* value for differences in BMI across both groups.

A STROBE checklist was completed to ensure the manuscript aligns with recommended methods for an observational study, and the checklist has been submitted as a supplement.

## 3 Results

**FIGURE 1:**
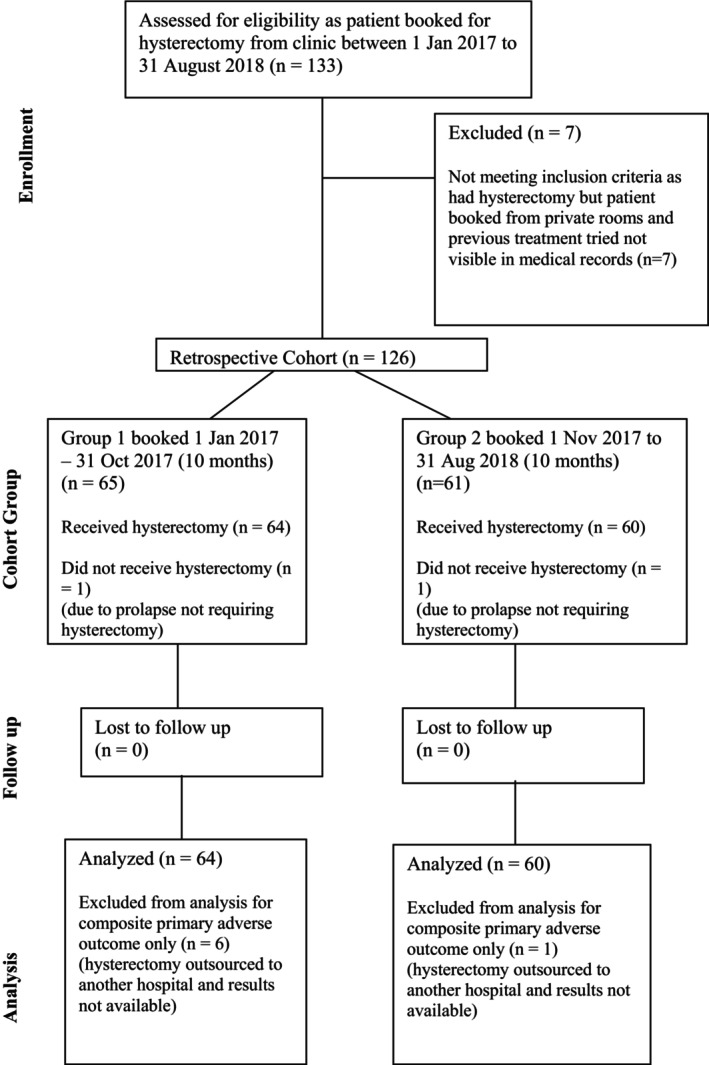
CONSORT diagram showing the flow of participants through the retrospective cohort study.

A total of 133 patients met the inclusion criteria. After exclusions, 64 patients were in Group 1 and 60 in Group 2. Please see Figure 1, which is a Consort flow diagram of eligible participants.

Table 1 describes overall demographic, procedure, and outcome data in the two groups, which show similar demographic data between the two groups, noting slight differences in BMI categories and more patients had a normal BMI in Group 2. Enrolled participants were considered similar, and no further matching criteria were applied.

**TABLE 1:** Demographics, indications, outcomes of benign hysterectomy.

	Group 1	Group 2	Difference in proportion Group 2‐Group 1	95% CI proportion difference, *p*
Total patients (*N*)	64	60		
Age mean (range) in years	47 (28, 81)	49 (28, 83)		
Age median	46	46		
Age SD	10	11		
**BMI category (*N*, %)** ^a^				
15–19.9	5 (7.9%)	2 (3.3%)	Chi square test 7.09	*p* = 0.069
20–29.9	24 (37.5%)	29 (48.3%)		
30–39.9	24 (37.5%)	25 (41.2%)		
40+	8 (12.7%)	1 (1.7%)		
**Mode of hysterectomy (*N*, %)**				
LAVH/LAVH‐BS	2 (3.1%)	0 (0%)	−0.0313	[−0.107, 0.033]
TAH/TAH‐BS	13 (20.3%)	16 (26.7%)	0.0635	[−0.086, 0.211]
TLH/TLH‐BS	34 (53.1%)	30 (50.0%)	−0.0313	[−0.201, 0.141]
VH/VH‐BS	15 (23.4%)	13 (21.7%)	−0.0177	[−0.163, 0.130]
Missing data (*N*, %)	0 (0%)	1 (1.7%)		
**Major indication (*N*, %)**				
Polyp	0	0	0	[−0.057, 0.060]
Adenomyosis	0	0	0	[−0.057, 0.060]
Leiomyoma	0	2 (3.3%)	0.033	[−0.028, 0.113]
Malignancy or hyperplasia	8 (12.5%)	2 (3.3%)	−0.917	[−0.197, 0.001]
Coagulopathy	0	0	0	[−0.057, 0.060]
Ovulatory dysfunction	0	0	0	[−0.057, 0.060]
Endometrial	31 (48.4%)	19 (53.3%)	0.0484	[−0.124, 0.218]
Iatrogenic	0	0	0	[−0.057, 0.060]
Not otherwise specified	9 (14.1%)	3 (5%)	−0.906	[−0.201, 0.018]
Post‐menopausal bleeding	3 (4.7%)	2 (3.3%)	−0.014	[−0.099, 0.073]
Pain	4 (6.3%)	7 (11.7%)	0.0542	[−0.051, 0.166]
Prolapse	9 (14.1%)	12 (20%)	0.0594	[−0.07, 0.194]
**Anaesthetic time (min)**				
Mean (SD)	137 (50)	145 (50)		
Range (minimum, maximum)	(37, 266)	(68, 302)		
**Length of stay (days)**				
Mean (SD)	2 (0.9)	2 (0.7)	^b^	
Range (minimum, maximum)	(1, 5)	(1, 6)		
Missing data (*N*)	6	1		
**Histopathology findings (no, %)** ^c^				
Polyp	2 (3.1%)	4 (6.7%)	0.0354	[−0.050, 0.131]
Adenomyosis	32 (50%)	29 (48.3%)	−0.0167	[−0.187, 0.155]
Leiomyoma	26 (40.6%)	26 (43.3%)	0.027	[−0.143, 0.200]
Malignancy or hyperplasia	1 (1.5%)	1 (1.7%)	0.001	[−0.074, 0.068]
Coagulopathy	0	0	0	[−0.057, 0.060]
Ovulatory	0	0	0	[−0.057, 0.060]
Endometrial	0	2 (3.3%)	0.033	[−0.028, 0.114]
Iatrogenic	0	0	0	[0.057, 0.060]
Not yet classified	11 (17.1%)	15 (25%)	0.0781	[−0.066, 0.221]
Pathology data incomplete	3 (4.6%)	0	−0.0469	[−0.029, 0.121]
Number with multiple findings on histopathology	12 (18.9%)	18 (30%)	0.1125	[0.039, 0.259]
**Contracted service (*N*, %)**	6, 9.4%	1, 1.7%		
**Intensive care unit (ICU) stay**				
Yes (*N*, %)	0	0 (0)	^b^	
No (*N*, %)	58	59 (98.3%)		
Missing (*N*, %)	6	1 (1.7%)		
**Blood transfusion**				
Yes (*N*, %)	1 (1.6%)	0 (0)	^b^	
No (*N*, %)	57 (89.1%)	59 (98.3%)		
Missing (*N*, %)	6 (9.4%)	1 (1.7%)		
**Readmission within 28 days including emergency department visit**				
Yes (*N*, %)	7 (10.9%)	4 (6.6%)	^b^	
No (*N*, %)	52 (81.2%)	55 (91.7%)		
Missing data (*N*, %)	5, (7.8%)	1, (1.7%)		
**Mortality**				
Yes (*N*, %)	0 (0%)	0 (0%)	^b^	
No (*N*, %)	63 (98.4%)	60 (100%)		
Missing (*N*, %)	1 (1.6%)	0 (0%)		
**Organ injury**				
Yes (*N*, %)	1 (1.6%)	1 (1.7%)	^b^	
No (*N*, %)	57 (89.1%)	58 (96.7%)		
Missing date (*N*, %)	6 (9.4%)	1 (1.7%)		
**Composite adverse outcome within 28 days (organ injury, readmission, ICU stay, mortality, blood transfusion, return to theatre)**				
Yes (*N*, %)	8 (12.5%)	4 (6.7%)	^b^	
No (*N*, %)	51 (79.7%)	55 (91.6%)		
Missing data (*N*, %)	6 (9.3%)	1 (1.7%)		
TAH/TAH‐BS with no prior caesarean and no cancer (broad definition low‐value care)	Total 6 (9.4%)	Total 7 (11.7%)	0.023	[−0.090, 0.140] *p* = 0.33

Abbreviations: BMI, body mass index; LAVH/LAVH‐BS, laparoscopic‐assisted vaginal hysterectomy without/with bilateral salpingectomy; SD, standard deviation; TAH/TAH‐BS, total abdominal vaginal hysterectomy without/with bilateral salpingectomy; TLH/TLH‐BS, total laparoscopic vaginal hysterectomy without/with bilateral salpingectomy; VH/VH‐BS, vaginal hysterectomy without/with bilateral salpingectomy.

^a^ Missing three patients for Groups 1 and 2.

^b^ Did not calculate 95% CI due to being unable to apply formula with missing data.

^c^ (histopathology) Not mutually exclusive.

The most common indication for hysterectomy was endometrial or dysfunctional uterine bleeding, comprising 48.4% and 53.3% of the primary indication for hysterectomy across Groups 1 and 2, respectively. The most common hysterectomy approach was laparoscopic.

There was a non‐significant increase in the low‐value hysterectomy rate 10 months following publication of the national HMB Standard, from 9.3% (6 patients) in Group 1 to 11.6% (7 patients) in Group 2, 95% CI [−0.09. 0.14], *p* = 0.33.

Regarding composite major adverse outcome including readmission, only those with complete data could be evaluated, which excluded six further patients in Group 1 and one patient in Group 2. Then, 8 out of 58 had a composite major adverse outcome in Group 1, compared to 4 out of 59 in Group 2. There were no mortality events.

Histopathology review revealed adenomyosis and leiomyoma were present in between 40% and 50% of specimens across both Groups 1 and 2. Stage 1 uterine cancer was diagnosed in 1.5% of Group 1 specimens and 1.7% of Group 2.

There were 54 patients booked for hysterectomy in Group 1 and 46 in Group 2 who received a hysterectomy with benign HMB as either their primary or secondary indication. Compliance to the HMB Standard was assessed and described in this group of patients in Table 2. Compliance improved across the audit period in only four out of eight standards. However, the two standards pertaining to specialist care improved.

**TABLE 2:** Hysterectomy for benign heavy menstrual bleeding: Comparison of Groups 1 and 2 in adherence to national Heavy Menstrual Bleeding Clinical Care Standard (HMB Standard) pre‐operatively.

	Group 1 before guideline publication January–October 2017	Group 2 after guideline publication November 2017–August 2018	Improvement in compliance from Group 1 to 2 (Yes or No)
Major or secondary indication was heavy menstrual bleeding	54 (100%)	46 (100%)	
Total denominator *N* (%)	30 (55.6%)	24 (52.1%)	No
Standard 2. Documented discussion on options for management with benefits and risks	34 (63.0%)	20 (43.5%)	No
Standard 3A. Offered tranexamic acid or non‐steroidal anti‐inflammatory drug	15 (27.8%)	10 (21.7%)	No
Standard 3B. Offered progesterone or combined oral contraceptive pill	17 (31.5%)	16 (34.8%)	Yes
Standard 4. Quality ultrasound days 5–10 of cycle	38 (70.4%)	40 (87.0%)	Yes
Standard 5A. Offered levonorgestrel intrauterine device (LNG‐IUD)	24 (44.4%)	14 (30.4%)	No
Standard 5B. Number who tried LNG‐IUD	22 (40.7%)	14 (30.4%)	No
Standard 6. Specialist referral if suspected malignancy, intrauterine pathology or after 6 months HMB with no improvement	43 (79.6%)	42 (91.3%)	Yes
Standard 7. Offered uterine conserving option such as ablation or focused surgical treatment	38 (70.4%)	34 (73.9%)	Yes
Standard 7A. Number of women who underwent endometrial ablation prior	5 (9.3%)	8 (17.4%)	Yes
Standard 8A. Hysterectomy is discussed when other options are ineffective, unsuitable, or at woman's request	37 (68.5%)	36 (78.3%)	Yes
Standard 8B. Hysterectomy risks and benefits clearly discussed and documented	50 (92.5%)	44 (95.7%)	Yes
Documents infection risk	43 (79.6%)	43 (93.4%)	Yes
Documents bleeding risk	43 (79.6%)	44 (95.7%)	Yes
Documents injury to organs, bladder, bowel, and ureter	42 (77.8%)	44 (95.7%)	Yes
Documents time in hospital and recovery	40 (74.1%)	43 (93.4%)	Yes

Regarding standards 3, 5, and 7, which concerned therapeutic alternatives to hysterectomy, all alternatives had compliance at less than 50% for each group, and there was a mix of both improvement and reduction in compliance. The proportion of patients who tried a levonorgestrel IUD before their benign hysterectomy for HMB was low and decreased from 40.7% in Group 1 to 30.4% in Group 2 (Standard 5B). The proportion of patients who tried endometrial ablation prior to hysterectomy for HMB was also low, although it increased from 9% in Group 2 to 17% in Group 1 (Standard 7B).

Table 3 shows the waiting time in days from referral to procedure of hysterectomy and various points of care in between. The wait times were similar for the two groups.

**TABLE 3:** Wait times.

	Group 1	Group 2
Total number of patients (*N*)	64	60
**Time from referral to clinic (days)**		
Median	44	78
IQR	53	202
Range (minimum, maximum)	(1, 521)	(4, 2649)
**Time from clinic visit to hysterectomy booked (days)**		
Median	87	50
IQR	261	162
Range (minimum, maximum)	(0, 1855)	(0, 1576)
**Time on surgical wait list (days)**		
Median	84	69
IQR	136	104
Range (minimum, maximum)	(0, 314)	(12, 538)
**Total wait from clinic referral to hysterectomy (days)**		
Median	215	197

*Note:* This section describes the waiting time between different dates listed in the data. The dates considered were the time from referral date to clinic visit date, the time from clinic visit to the date booked for hysterectomy, and the time from the date booked for hysterectomy to the operation date. All times are presented in days. For times, generally the median is preferable to report as it is not impacted by extreme values.

## 4 Discussion

### 4.1 Statement of Principal Findings

This study found no significant change in low‐value hysterectomy in the 10 months post‐publication of the 2017 HMB Standard in an Australian regional health service, compared to the 10 months prior.

In assessing our secondary aim, we found mixed results regarding compliance with the HMB Standard. Notably, the standards around therapeutic alternatives to hysterectomy had overall low compliance across both groups.

### 4.2 Strengths and Weaknesses of the Study

A strength of our study is that we specifically evaluated value‐based care in a non‐urban hospital setting, where previously there has been a lack of research [9, 19].

Additionally, the findings from our study have identified local gaps in care that has prompted unit improvement work.

Regarding limitations, our findings are not generalisable as we focused on a single hospital in regional Victoria. Further, our analysis is largely descriptive, which, while an important part of epidemiological research, limits our ability to understand the impact of any potential confounding factors or draw any causal conclusions.

Another limitation was that there was a delay in the completion of this project with the challenges to the healthcare workforce in this Victorian regional hospital, which means this project is reflecting data from 6 years ago. Australian data have reflected a reduction in benign hysterectomy since this time, as published in the latest Atlas report [15].

### 4.3 Strengths and Weaknesses in Relation to Other Studies

Our study faced similar limitations in evaluating hysterectomy variation as other reported projects evaluating hospital data, in that we were reliant on what was captured in the patient records [20]. Although we manually reviewed records, unlike other larger studies that were able to [10], we were still limited by what was documented at the time.

Previous Australian studies on low‐value hysterectomy have looked at larger patient numbers and could have more generalisable results than our study [11, 20].

### 4.4 Meaning of the Study

This study highlights several areas for improvement in standardising access to less invasive options recommended in the HMB Standard in our regional centre [14, 15].

The findings from our study have prompted the introduction of a surgical planning meeting where all patients planning hysterectomy under 35 years of age or with medical, anaesthetic, or surgical complexity are reviewed by a multi‐disciplinary panel to ensure appropriate assessment and planning. We have also commenced an HMB clinic with a focus on therapeutic alternatives to hysterectomy in accordance with the 2024 HMB Guideline [15]. Other authors have found that local clinician‐led interventions are an effective method to reduce low‐value care [20]. We look forward to reviewing the impact of these changes on low‐value hysterectomy rates in our hospital in the future.

### 4.5 Unanswered Questions and Future Research

Our study has not answered why the patients were not offered or did not try therapeutic alternatives to hysterectomy. It would be interesting to survey patients and clinicians as to whether there was adequate training provided to clinicians, adequate time allocated in clinics, what information patients received, and whether patients perceived access to primary care as a driver.

It is unclear how much the regional location of the patients impacted their care. The Atlas reported that non‐urban patients were more likely to have benign hysterectomy [12] and it would be interesting to further explore socio‐economic and geographical factors that may be contributing to this.

## Author Contributions

**Natasha Daureen Frawley:** conceptualization, methodology, investigation, data curation, writing – original draft, reviewing and editing. **Madison Phung:** investigation, data curation, writing – reviewing and editing. **Benjamin Harrap:** methodology, formal analysis, writing – reviewing and editing.

## Ethics Statement

Ethics approval was obtained by the SJOGGH research ethics department, project number 44872, obtained on 19 December 2019.

## Conflicts of Interest

The authors declare no conflicts of interest.

## Data Availability

The data that support the findings of this study are available from the corresponding author upon reasonable request.
